# Presidential Election 2022: A Euroclash Between a “Liberal” and a “Neo-Nationalist” France Is Coming

**DOI:** 10.1007/s10272-021-0957-x

**Published:** 2021-04-06

**Authors:** Thierry Chopin, Samuel B. H. Faure

**Affiliations:** 1 European School of Political and Social Sciences, Catholic University of Lille (ESPOL), 60 boulevard Vauban, CS 40109, 59016 Lille Cedex, France; 2 Political Science, Sciences Po Saint-Germain-en-Laye, 5, rue Pasteur, 78100 Saint-Germain-en-Laye, France

## Abstract

The main question surrounding the re-election of Macron is the uncertain balance of France’s flexilateral policy vis-à-vis the EU in a post-Brexit and COVID-19 context: Strengthening European integration by changing France’s practice towards the EU or taking an inter-governmentalist turn by using – in a very classical way – Europe as an “Archimedes’ lever” to defend national interests.

How could the 2022 French presidential election impact the dynamics of European integration? Generally speaking, there is an increasingly strong link between national elections and domestic politics on the one hand and European issues on the other (Bulmer and Lequesne, 2020). From the point of view of the national context in France, the presidential political system gives major importance to this election because the most strategic decisions with European partners are still made by the president – all the more so in a context of crises that reinforce the role of the European Council (Wessels, 2015). The EU has been marked since the beginning of the 21st century by a "polycrisis" (Juncker, 2016) that has strengthened the institutional position of the European Council (Bickerton et al., 2015). Moreover, the current COVID-19 crisis poses a number of challenges to the citizens of EU member states that have a clear European dimension, such as economic recovery, energy transition, defence policy, etc.

**Thierry Chopin**, Université Catholique de Lille, ESPOL, France.

Given the high level of uncertainty at this political moment (as it is difficult to know to what extent we will still be in crisis or post-crisis at the beginning of the election campaign in less than a year) as well as the electoral volatility and the "crisis of democracy" at the national level, we have to be cautious in the following analysis. Drawing on recent research conducted in European studies, this article develops a typology of the five models of French political actors' positions vis-à-vis the EU: the "left-wing sovereignist", the "social-economic integration", the "liberal integration", the "right-wing sovereignist" and the "neo-nationalist" models. This typology is constructed by cross-referencing the ideological preferences of political leaders and their choices regarding European governance. By taking into account both structural trends and the political situation informed by polls, we develop two scenarios corresponding to the re-election of Emmanuel Macron and the election of Marine Le Pen. The first scenario embodies the liberal model, while the second defends the neo-nationalist model, but both are distinguished by their preferences for the integration and differentiation of EU governance, the level of politicisation of European bargaining and priorities of the political agenda. These differences outline two dynamics of Europe in the first part of the 21st century.

## The making of French politics through ideological preferences and European governance

French politics are in a process of strong evolution, determined by the "new public management", which stems from technocracy and expertise, and aims at the imperative of "efficiency" (Schmidt and Thatcher, 2013). The authority of the head of state and the permanent link between the head of state and the people are reinforced by the presidentialist institutions of the Fifth Republic: It is the "return of the Prince" (Martigny, 2019). The omnipresence of social media shakes up public debate by reinforcing the salience of certain public problems such as *laïcité*, or secularism. France is thus defined by a new logic of democracy in Europe described as "techno-populism" (Bickerton and Invernizzi Accetti, 2021). Marine Le Pen (Rassemblement National, RN) and Emmanuel Macron (La République en Marche, LREM), who reached the second round of the presidential election in 2017 and have dominated the polls ever since, present themselves as anti-establishment[1] and defend a policy of radical reforms.[2] Le Pen and Macron see themselves as outsiders in French politics embodying a new era (*nouveau monde*).

However, Le Pen has been a major player since the turn of the century (MEP and regional councillor since 2004), and Macron was adviser (2012-14) and then Minister of the Economy (2014-16) under the mandate of his predecessor, President François Hollande. But above all, this political opposition between "techno-populists" has not replaced the traditional ideological divide between the left and the right, which is proving resilient,[3] even within LREM. The left-right rivalry is characterised, on the one hand, by the nature and degree of political regulation chosen by the actors and, on the other hand, by their framing of the political agenda (Table 1). This positioning can be analysed using the relations with liberalism: In France, the left is liberal politically and culturally but to a large extent opposed to economic liberalism; the right is liberal economically but not culturally, prioritising security and traditional values over individual liberties; and the extremes are illiberal politically and economically.

French left-wing actors defend a strong market interventionism with "dirigiste" capitalism, while being more liberal in their vision of society regarding, among other things, minority rights and youth. On the other hand, right-wing actors value a weaker regulation of the market by a state with reduced prerogatives and (neo)-liberal economic logic, while supporting conservative positions on societal issues.[4] Moreover, left-wing programmes are oriented around climate change, the ideal of social justice and equality for all citizens, as well as institutional transformations in favour of a more participatory and therefore bottom-up democracy. Conversely, French right-wing actors put the competitiveness of businesses and the value of freedom, the issue of national identity linked to security, and the status quo of a centralised and top-down organisation of the political regime on the political agenda.

Table 1
**The left-right divide in French politics**

| | | Left | Right |
|---|---|---|---|
| State regulation | Market regulation | High | Low |
| | Role of public authorities | High | Low |
| | Regulation of civil society | Low | High |
| Policy framing | Economic and social issues | Equality and solidarity | Business competitiveness and freedom |
| | Societal issues | Climate change and justice | National identity and security |
| | Institutional issues | Change | Statu quo |

Source: Authors' own elaboration.

This left-right paradigm is useful but insufficient to capture the preferences of French actors linked to European politics, often summarised in public debate by a rivalry between the "sovereignist" camp, which defends the nation, and the "globalist" camp, which values the EU (Grunberg, 2021).[5] Research conducted on EU member states demonstrates that it is more heuristic to make a distinction in terms of governance (Bulmer and Lequesne, 2020). Some French actors favour a strictly intergovernmental functioning of the EU. They prefer the European Council and the Council of Ministers, arenas where decisions are taken by unanimity (Novak, 2017). They are inclined to politicise negotiations, use their veto to defend national interests and block positions preferred by other member states. Within and beyond EU institutions, the effort to create more political opposition to the EU is labelled "Euroclash" (Fligstein, 2008) and can lead to institutional crises such as the "empty chair" crisis in the 1960s, which concerned the common agricultural policy (Moravcsik, 1998).

1 They share the "*dégagisme*" idea that French political problems would come from a unified political class around outdated political parties, in particular, the Parti Socialiste on the left and Les Républicains on the right.
2 Macron (2016) published a book entitled *Revolution* during the election campaign.
3 It may be recalled that Le Pen and Macron represent less than one out of two voters. Moreover, a significant proportion of citizens, sometimes a majority of them as in the European elections of 2019 and the municipal elections of 2020, do not vote, confirming the argument of a "democracy of abstention" (Braconnier and Dormagen, 2007).
4 This assertion should not obscure the fact that there is a tradition of a Colbertist right in France, which defends state interventionism vis-à-vis the market.
5 We find this cross-party polarisation in other international issues such as France's position vis-à-vis Russia (Schmitt, 2017) or the United States (Charillon, 2021).

Other French leaders do not limit themselves to intergovernmental work in order to govern the EU, but also involve supranational institutions such as the Commission, the European Parliament, the Court of Justice and the European Central Bank (ECB).[6] Supranational institutions are not abandoned or criticised – their political competences being delegitimised in the name of national sovereignty – but are conversely used as another political instrument to promote a "French Europe" (Rozenberg, 2020). In the context of a differentiated European integration (Chopin, 2017a; Faure and Lebrou, 2020; Schimmelfennig and Winzen, 2020), the articulation by a state of several formats (bi-, mini- and multilateral), arenas (within and outside the EU) and instruments (intergovernmental and supranational) of European cooperation can be called "flexilateralism" (Faure, 2019a).

To summarise and reformulate it in a more conceptual way (Bickerton, 2012), while Macron favours France's link to the EU and a consensual practice of power (i.e. the "member state model"), Le Pen could choose "the people against Europe" by reinforcing the politicisation of the way France's European policy is conducted (i.e. the "nation state model").

Figure 1
**Five positions of French political actors vis-à-vis the EU**

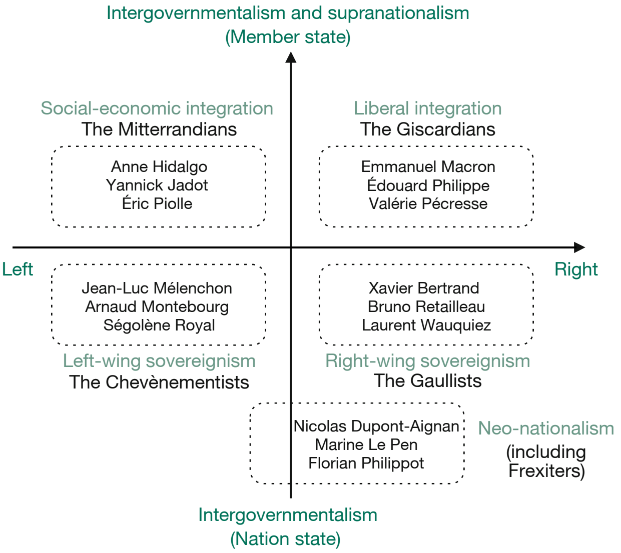

Source: Authors' own elaboration.

## Mapping French political actors' positions on EU politics

The intersection of these two ideological and political continua reveals the distribution of French actors between five models of political position vis-à-vis the future of European integration (Figure 1). Like any typology, this one does not exhaust the complexity of reality. However, this typology shows the main political trends that order the national political field vis-à-vis the EU and can thus improve our understanding of how European issues will be addressed in the 2022 presidential election.

The first group brings together actors who defend a left-wing political agenda and values by supporting strong state interventionism and intergovernmental European governance: This is the "left-wing sovereignist" model regrouping as the "Chevènementists". Jean-Luc Mélenchon (La France Insoumise), as well as certain former members of the Parti Socialiste, such as Ségolène Royal and Arnaud Montebourg, are the heirs of this model. Indeed, Royal had oriented her 2007 presidential campaign (which she lost in the second round to Nicolas Sarkozy) around the ideas of nation, sovereignty and borders. Arnaud Montebourg (MP, 1997-2012; Minister of the Economy, 2012-14) has positioned himself since the beginning of 2010 as the main promoter of "Made in France" and thus of an assumed economic protectionism.

A second cluster of political actors is identified on the left and differs from the first one by defending a more integrated EU governance: This is the "social-economic integration" model associated with the "Mitterrandians". This political line is embodied by the ecologist party (Europe Écologie-Les Verts) and its main representatives, such as MEP Yannick Jadot and the Grenoble mayor Éric Piolle. The Socialists' contingent who voted in favour of the Treaty establishing a Constitution for Europe (TCE) in 2005 share this political orientation towards a deeper European integration. This is the case of the former President, François Hollande, as well as his Minister of Justice, Christiane Taubira, and the current mayor of Paris, Anne Hidalgo. Without clearly establishing the links between the political and intellectual fields, this position corresponds to the proposals formulated by Thomas Piketty among others (Hennette et al., 2019) in favour of a treaty for the democratisation of Europe. According to them, the main institutional challenge is not so much safeguarding national sovereignty, but rather crafting a transnational democracy.[7]

6 The federalist movement that would lead political parties to defend an exclusively supranational governance of the EU is absent from the French political landscape, being reduced to a few isolated figures such as Daniel Cohn-Bendit, who no longer play an active role.

7 This also recalls the legal and economic contribution of Aglietta and Leron (2017), as well as the recent tribune of Vallée (2020), who also insists on the democratic stake.

The third guild of French leaders shares support for a flexilateral EU (intergovernmental and supranational governance) with the previous group while distinguishing itself with a preference for centre-right political ideas and values: This is the "liberal integration" model that unites the "Giscardians".[8] The European policy conducted by President Macron since 2017 and by the former Prime Minister Édouard Philippe (2017-2020) corresponds to this model, which does not seem incompatible with the moderate part of the conservative party (Les Républicains) embodied by Valérie Pécresse, president of the Île-de-France region.

The fourth model of political actors, like the previous group, favours a preference for right-wing political values, but moves away from it with a strictly intergovernmental governance of the EU: This is the "right-wing sovereignist" model that binds the "Gaullists". It associates part of Les Républicains, such as Bruno Retailleau, Senator since 2004 and President of the Les Républicains group in the Senate since 2014, and also Laurent Wauquiez and Xavier Bertrand, presidents of the Auvergne-Rhône-Alpes and Hauts-de-France regions, respectively.[9] Their political positions on security and immigration converge, as does a defence of the rural society and traditions (Rozenberg, 2020, 82).

Finally, a fifth model involves the "neo-nationalists" (Badie et al., 2017). Unlike the two classic types of nationalism ("liberal" nationalism linked to the principle of self-determination of peoples and "authoritarian and expansionist" nationalism which marked the history of the first half of the twentieth century), the return of nationalism nowadays (neo-nationalism) is more specifically a nationalism of withdrawal and protection which is characterised by a defensive political discourse. Neo-nationalist movements take advantage of the context of the current sovereignist moment (Lamassoure et al., 2019).

This neo-nationalist model includes political leaders who defend France's exit from the EU (Frexit). This position has been residual in the political arena since Le Pen changed her narrative and strategy when she lost the presidential election in 2017, stating that France would remain within the EU and the eurozone if she were elected President of the French Republic. Frexit is supported by far-right micro political parties, such as Les Patriotes led by Florian Philippot. The latter was Le Pen's closest collaborator between 2012 and 2017 as vice president of the Front National (FN). Other movements include Debout la France led by the MP Nicolas Dupont-Aignan and François Asselineau's Union populaire républicaine (UPR).

Table 2
**A comparison between Macron and Le Pen programmes vis-à-vis the EU**

| | Macron | Le Pen |
|---|---|---|
| Political position vis-à-vis the EU | Liberal model | Neo-nationalist model |
| Type of state | Member state | Nation state |
| Level of governance | European | National |
| Level of integration | High | Low |
| Level of differentiation | High | High |
| Level of politicisation | Low | High |

Source: Authors' own elaboration.

While no extreme left-wing political movement has openly pronounced itself in favour of Frexit, an ideological and semantic shift is observed among some leaders of La France Insoumise. For example, Jean-Luc Mélenchon stated on Twitter on 30 January 2020, the day Brexit was activated: "The United Kingdom, freed from the tutelage of Brussels, is renationalising the railroad that the liberals had put into chaos. Independence is paying off". In the context of social inequalities increased by the COVID-19 pandemic, it would not be surprising if an activist from the yellow vests social movement were to present him- or herself in 2022 on a similar political line.

Given the current polls (Gallard et al., 2021), more than a year before the presidential election, the following two sections focus on two of these five models forming a Euroclash: the liberal model represented by Macron's re-election and the neo-nationalist model embodied by the election of Le Pen. For each scenario, we analyse the political and institutional preferences for governance, as well as the political agendas related to the main economic and social issues (Table 2).

## Macron's re-election: An uncertain idea of France as a member state

The main question surrounding the re-election of Macron is the uncertain balance of France's flexilateral policy vis-à-vis the EU in a post-Brexit and COVID-19 context: Strengthening European integration by changing France's practice towards the EU or taking an inter-

8 The difference between social-economic integration and liberal integration models corresponds, conceptually (Scharpf, 1999), to the distinction between "positive integration" (market correcting) and "negative integration" (market making).

9 Although Laurent Wauquiez and Xavier Bertrand voted in favour of the TCE in 2005, Wauquiez considered a posteriori that he had changed his mind by taking a sovereignist turn developed in his 2014 book and Bertrand spoke out against the Maastricht Treaty in the referendum organised in 1992.

governmentalist turn by using – in a very classical way – Europe as an "Archimedes' lever" to defend national interests. In the hypothetical case of Macron's re-election, it seems clear that the President of the French Republic will seek to pursue the implementation of his agenda aimed at developing the constitution of European sovereignty (Macron, 2017; Beaune, 2020). This project is not perceived as the will to build a federal state at the European scale, but to consider that the EU constitutes a relevant scale of public action complementary to the national level of the member states in order to provide responses to challenges that both go beyond nations and concern the heart of state sovereignty (Chopin, 2017b).

Nonetheless, this notion of European sovereignty presents a certain ambivalence (Bertoncini and Chopin, 2020a, 2020b; Fondation Jean Jaurès and Fondation Friederich-Ebert, 2021). It leads to the defence of the Mitterrandian-Delorist position reflecting a preference for a deeper socio-economic integration, and at the same time, working in favour of a Gaullist and/or a Chevènementist position that supports national sovereignty (Faure, 2020a). In the COVID-19 context, we observe a semantic shift from the theme of sovereignty to that of independence, "Made in France" and national sovereignty over European sovereignty, and strategic autonomy (Faure, 2020b; Tertrais, 2021). The discourse carried by Macron is increasingly similar to the traditional French narrative that France should use the EU as a power multiplier, the famous Archimedes' lever referred to by General Charles de Gaulle himself. In other words, Macron shares with his predecessors a "French Europe", i.e. a "certain idea of Europe" (Parsons, 2006). This could be summed up by considering that Macron's European policy is featured both by a certain idea of France (Jackson, 2018) and by an uncertain idea of Europe (Faure, 2020c).

This political ambiguity is reflected in institutional issues. Is it a question of strengthening the role of the European Commission and Parliament in the political governance of the EU? Or rather promoting European governance foremost through the European Council and the role played by the heads of state and government, a role that has been strongly reinforced by the ten years of "polycrisis" that have affected Europeans? The French President seems to favour voluntarism and the search for leadership rather than the patient search for a compromise negotiated with his European partners, which corresponds to French institutional and political practice and habits (Chopin, 2017b). This shows a preference for the European Council as the real executive power of the EU and an intergovernmental conception of European policy. Nevertheless, this political style seems to be combined at the same time with a focus on supranational institutions (and France's influence within them), especially the Commission, the Parliament and the ECB (Bertoncini and Chopin, 2019).

Concerning the programme and the political preferences likely to be carried in the next presidential election, they should be as high on the candidate's strategy and agenda as in 2017, for at least three reasons. First, Europe is at the heart of the political DNA of Macron's voters. Second, the left and the right are divided on the European issue. Third, in the perspective of a "return match" against Le Pen, Europe will be a very divisive issue politically, notably in the context of the French Presidency of the EU Council. With regard to this last point, however, it should be noted that the terms of the debate will not be the same in 2022 as they were in 2017. On the one hand, Macron will have to defend the European assessment of his five-year term; on the other hand, Le Pen has abandoned the Europhobic "exit" strategy and has refocused her speech on the classic themes of the extreme right, such as immigration and security. Moreover, the question arises as to whether the French President will seek to pursue the promised "European Renaissance" or whether he will decide to promote a less ambitious programme with the choice of a few (more) modest reforms. Finally, the themes and main features of the 2022-27 programme could be defined on the basis of the priorities that have been announced for the forthcoming French Presidency of the EU Council in the form of the triptych: *relance* (recovery), *puissance* (power) and *appartenance* (belonging). This discursive register and political agenda seems to confirm and extend the liberal model mentioned above.

### Le Pen's election: A demanding remainer in favour of the nation state

If we know that Le Pen has renounced her "exit" strategy and the will to leave the EU, her position once she comes to power will be neo-nationalist, corresponding to the slogans "Make France Great Again" and "France First". In such a perspective, Le Pen would support national sovereignty rather than European sovereignty and the preference (when possible) for ad hoc bilateral or minilateral collaborations rather than the use of the multilateral framework within the EU. The question arises as to what would happen in a situation of cohabitation if the RN does not manage to obtain an absolute majority in the legislative elections following the presidential election: Would this lead to a paralysis of Europe by a regime crisis? Alternatively, what would happen without cohabitation? Would it lead to a Frexit by referendum, even if opinion polls clearly indicate the absence of a majority Europhobia, or hard Euroscepticism, in France (see Chopin and Lequesne, 2020; Chopin et al., 2020b)?

In such a scenario, the election of Le Pen would first of all have a number of obvious implications in terms of European governance. France would undoubtedly favour a strict intergovernmental functioning in accordance with a conception of the EU as an alliance of sovereign national states seeking above all else to defend their national interests, i.e. the Gaullist model. It should be stressed that such a practice of power at the European level could risk blockages, or even paralysis, in the event of too strong cohabitation and confrontation between the president and the prime minister or certain powerful sectoral ministers from another political party. Alternatively, one could imagine a duplicitous game between the "moderates" and the "radicals" – as among the Tories between David Cameron and Boris Johnson (Evans and Menon, 2017) – with a sectoral minister who would push the president further to the right, and further away from the EU, to the point of asking for a referendum. Furthermore, it is likely that the presidency of Le Pen will seek to cooperate in Europe as much as possible outside the EU through ad hoc cooperation, and moreover bilaterally, where Macron would continue to play first and foremost the EU game (Table 2). This would lead to the strengthening of the logic of differentiation of Europe not so much as a path to integration which has been a structuring logic in the history of European construction (Chopin and Jamet, 2008; Faure, 2019b), but rather leading the EU towards the risk of fragmentation and even of disintegration (Jones, 2018; Webber, 2018).

The election of Le Pen could change the practice of governance in the EU and France's influence accordingly. On the political level, it is possible to anticipate a reinforcement of the intergovernmental politicisation of the negotiations in the European Council, following Viktor Orban's practices to push the comparative advantage to the breaking point. Moreover, the political credibility of France, a "large" founding country participating in all EU policies (the euro, Schengen, etc.), could be strongly affected. It is difficult to imagine that Le Pen, elected to the Presidency of the Republic, would be able to exercise political leadership at the European level with France's partners in a context where the health crisis will also have reduced its budgetary room for manoeuvre, thus reducing the country's economic credibility. On the institutional front, it is likely that Le Pen's strategy for France will lead to criticism of the supranational institutions (against the Commission of which the liberal Thierry Breton will still be a member until 2024, but also against the Court of Justice). In other words, while remaining within the EU, as already mentioned, we would observe the transition of France from a member state to a nation state (Bickerton, 2012).

In terms of a political agenda, Le Pen's strategy is to reassure and win over a "popular" right-wing electorate, particularly pensioners, whose voter turnout is traditionally high. On the economic front, the objective will be to propose a more reassuring economic programme. This would involve abandoning the most radical positions concerning Europe, which would bring uncertainty, particularly with regard to the exit from the euro, an abandonment that had already been noted at the time of the last European elections. The "moderation" of the economic discourse also involves a change in the thinking on debt with the abandonment of the idea of having the ECB cancel public debt. On the contrary, Le Pen has recently declared herself in favour of debt repayment while insisting on points already mobilised by the current government such as investments in the future, support for companies, etc. This economic narrative is keen to reassure right-wing voters who are attached to the conservation of their heritage. On the question of borders, it is likely that Le Pen develops a defensive and closed vision of European national societies in line with a neo-nationalist conception and advocates the closure of borders to immigration as well as the limitation of the free movement of people within the EU, including free movement within the Schengen area.

## Conclusions

Given the complexity of these issues and the contingency of the political situation, it would not be surprising if our probabilities do not hold. Perhaps Macron will not run again for the presidency, maybe Le Pen will not win. Moreover, this analytical grid is a draft, by definition imperfect and non-exhaustive. However, there is little doubt that the 2022 presidential election will be animated by a Euroclash, and we have explained the reasons that lead us to believe that this Euroclash will see the opposition of a liberal project to a neo-nationalist project, both situated on the right wing. Finally, we hope to have proposed an effective compass that will allow us to grasp the main political and institutional trends shaping French EU policy and which will undoubtedly impact the future of European integration.

## References

[CR1] Aglietta, M. and N. Leron (2017), *La double démocratie. Une Europe politique pour la croissance*, Le Seuil.

[CR2] Badie, B. and M. Foucher (2017), *Vers un monde neo-national?*, CNRS Editions.

[CR3] Beaune, C. (2020), L’Europe, par delà le Covid-19, *Politique étrangère*, 85(3).

[CR4] Bertoncini, Y. and T. Chopin (2019), Le choix des gouvernants de l’Union. Pour un meilleur équilibre entre démocratie et diplocratie, *Le Grand Continent*, https://legrandcontinent.eu/fr/2019/11/20/le-choix-des-gouvernants-de-lunion-pour-un-meilleur-equilibre-entre-democratie-et-diplocratie/ (15 March 2021).

[CR5] Bertoncini Y, Chopin T (2020). Macron l’Européen: de l’Hymne à la joie à l’embarras des choix. Le Débat.

[CR6] Bertoncini, Y. and T. Chopin (2020b), La “FrancEurope” 70 ans après la déclaration Schuman: projet commun ou projection nationale? (I–II), *Le Grand Continent*, https://legrandcontinent.eu/fr/2020/05/08/franceurope-declaration-schuman-chopin-bertoncini/ (15 March 2021).

[CR7] Bickerton, C. (2012), *European Integration: From Nation-States to Member States*, Oxford University Press.

[CR8] Bickerton C J, Hodson D, Puetter U (2015). The New Intergovernmentalism. European Integration in the Post-Maastricht Era. Journal of Common Market Studies.

[CR9] Bickerton, C. J. and C. Invernizzi Accetti (2021), *Technopopulism. The New Logic of Democratic Politics*, Oxford University Press.

[CR10] Braconnier, C. and J.-Y. Dormagen (2007), *La démocratie de l’abstention. Aux origines de la démobilisation électorale en milieux populaires*, Gallimard.

[CR11] Bulmer, S. and C. Lequesne (2020), *The Member States of the European Union*, Oxford University Press, Third Edition.

[CR12] Charillon, F. (2021), *La France dans le monde*, CNRS.

[CR13] Chopin, T. and J.-F. Jamet (2008), La différenciation peut-elle contribuer à l’approfondissement de l’intégration communautaire?, *Question d’Europe*, 106–107, Fondation Robert Schuman.

[CR14] Chopin T (2017). Après le Brexit: Vers une ‘Europe à trois vitesses’. Cités.

[CR15] Chopin, T. (2017b), Defending Europe to defend Real Sovereignty, *Jacques Delors Institute Policy Paper*, 194.

[CR16] Chopin, T. (2017c), Emmanuel Macron, la France et l’Europe. Le “retour de la France en Europe. A quelles conditions?”, in R. Brizzi and M. Lazar (eds.), *La France d’Emmanuel Macron*, Presses Universitaires de Rennes.

[CR17] Chopin, T. and C. Lequesne (2020a), Disintegration reversed: Brexit and the cohesiveness of the EU27, *Journal of Contemporary European Studies*, 13(1).

[CR18] Chopin T., B. Cautrès and E. Rivière (2020b), Les Français et l’Europe. Entre défiance et ambivalence, *Rapport*, 119, CEVIPOF-Sciences Po, Institut Jacques Delors, Kantar.

[CR19] Evans, G. and A. Menon (2017), *Brexit and British Politics*, Polity Press.

[CR20] Faure S B H (2019). Varieties of international co-operation: France’s ‘flexilateral’ policy in the context of Brexit. French Politics.

[CR21] Faure S B H (2019). The choice for a minilateral Europe. A historical sociology of defence-indusrial capitalism. European Review of International Studies.

[CR22] Faure, S. B. H. (2020a), French military strategy under Macron, in R. Johnson and J. Haaland Matlary (eds.), *Military Strategy in the 21st Century. The Challenge for NATO*, Hurst.

[CR23] Faure, S. B. H. (2020b, 15 June), Plan aéronautique: le “Made in France” prend le pas sur la préférence européenne, *HuffPost*, https://www.huffingtonpost.fr/entry/plan-aeronautique-le-made-in-france-prend-le-pas-sur-la-preference-europeenne_fr_5ee77734c5b6b41661031c75?utm_hp_ref=fr-homepage (15 March 2021).

[CR24] Faure, S. B. H. (2020c), Une idée incertaine de l’Europe. Comprendre les ambiguïtés stratégiques d’Emmanuel Macron, *Les Champs de Mars*, forthcoming.

[CR25] Faure S B H, Lebrou V (2020). L’Europe à géométrie variable. Renouveler l’analyse des logiques de différenciation de l’intégration européenne. Politique européenne.

[CR26] Fligstein, N. (2008), *Euroclash. The EU, European Identity and the Future of Europe*, Oxford University Press.

[CR27] Fondation Jean Jaurès, Fondation Friederich-Ebert (2021), De la souveraineté européenne, https://jean-jaures.org/nos-productions/de-la-souverainete-europeenne (15 March 2021).

[CR28] Gallard, M., B. Teinturier and S. Zumsteeg (2021), Présidentielle 2022: Macron et Le Pen favoris, mais Xavier Bertrand ou un candidat de la gauche unie seraient en position de créer la surprise, *Ipsos*, https://www.ipsos.com/fr-fr/presidentielle-2022-macron-et-le-pen-favoris-mais-xavier-bertrand-ou-un-candidat-de-la-gauche-unie (15 March 2021).

[CR29] Grunberg, G. (2021), Présidentielle 2022: tous souverainistes!, *Telos*, https://www.telos-eu.com/fr/politique-francaise-et-internationale/presidentielle-2022-tous-souverainistes.html (15 March 2021).

[CR30] Hennette, S., T. Piketty, G. Sacriste and A. Vauchez (2019), *How to Democratize Europe*, Harvard University Press.

[CR31] Jackson, J. (2018), *A Certain Idea of France. The Life of Charles de Gaulle*, Penguin.

[CR32] Jones E (2018). Towards a theory of disintegration. Journal of European Public Policy.

[CR33] Juncker J.-C. (2016), Conclusions of the European Council meeting of 17 and 18 December 2015, Speech at the EP Plenary session, https://ec.europa.eu/commission/presscorner/detail/en/SPEECH_16_112 (15 March 2021).

[CR34] Lamassoure, A., T. Chopin, P. Lamy, C. Verger, J.-L. Bourlanges, S. Maillard, G. Pons, R. Bloj ∣ C. De Nuzzo and G. Gressani (2019), Nationalisme, In Les mots de la campagne, Jacques Delors Institute and Le Grand Continent https://institutdelors.eu/en/publications/les-mots-de-la-campagne-le-nationalisme/ (15 March 2021).

[CR35] Macron, E. (2016), *Révolution*, XO.

[CR36] Macron, E. (2017), Initiative for Europe. A sovereign, united, democratic Europe, Speech at the Sorbonne University, Paris, 26 September 2017, http://www.elysee.fr/assets/Initiative-for-Europe-a-sovereign-united-democratic-Europe-Emmanuel-Macron.pdf (15 March 2021).

[CR37] Martigny, V. (2019), *Le Retour du Prince*, Flammarion.

[CR38] Moravcsik, A. (1998), *The Choice for Europe. Social Purpose and State Power from Messina to Maastricht*, Cornell University Press.

[CR39] Novak, S. (2017), La prise de décision dans l’Union européenne, in O. Costa and F. Mérand (eds.), *Études européennes*, Bruylant, 55–94.

[CR40] Parsons, C. (2006), *A Certain Idea of Europe*, Cornell University Press.

[CR41] Philippot, F. (2018), *Frexit. UE: en sortir pour s’en sortir*, L’artilleur.

[CR42] Rozenberg, O. (2020), ‘France is back’… in a French Europe, in S. Bulmer and C. Lequesne (eds.), *The Member States of the European Union*, Oxford University Press, Third Edition, 73–100.

[CR43] Schimmelpfennig, F. and T. Winzen (2020), *Ever Looser Union? Differentiated European Integration*, Oxford University Press.

[CR44] Scharpf, F. (1999), *Governing in Europe: Effective and Democratic?*, Oxford University Press.

[CR45] Schmidt, V. A. and M. Thatcher (2013), *Resilient Liberalism in Europe’s Political Economy*, Cambridge University Press.

[CR46] Schmitt, O. (2017), *Pourquoi Poutine est notre allié? Anatomie d’une passion française*, Hikari éditions.

[CR47] Tertrais, B. (2021), The Making of Macron’s Worldview, *World Politics Review*, https://www.worldpoliticsreview.com/articles/29362/how-france-s-jean-pierre-chevenement-shaped-the-macron-agenda (15 March 2021).

[CR48] Vallée, S. (2020), L’Union par la gauche, *Le Grand Continent*, https://legrandcontinent.eu/fr/2020/12/18/bilan-europe-macron/ (15 March 2021).

[CR49] Wauquiez, L. (2014), *Europe: il faut tout changer*, Odile Jacob.

[CR50] Webber, D. (2018), *European Disintegration? The Politics of Crisis in the European Union*, Palgrave Macmillan.

[CR51] Wessels, W. (2015), *The European Council*, Palgrave Macmillan.

